# Locations for Recent Graduates

**Published:** 1911-10

**Authors:** 


### Locations for Recent Graduates

THE general advice has often been given, to locate where one would be willing to spend one's life. On the other hand, recognizing the powerful leverage of money and the desirability of making the most active years of life as productive as possible, it is of advantage to the majority of young physicians who must regard their profession as a means of earning a living, that they should choose a location which shall, practically immediately, yield a living income. The choice of a thoroughly desirable neighborhood, in a city, involves, according to average experience, an income about equal to that of an office boy for the first year and it usually requires about four years to build up a practice that will insure a comfortable, plain living for a single man, or the bare necessities of life for a married man.

It has often seemed that the physician who acquires self-possession, practical experience, and a little capital and reputation in one place and who then goes to another, especially with some definite appointment, in the long run, makes the greatest practical success. On the other hand, the man who changes his location late in life, especially from a small to a large town, usually fails, so that the change, if made at all, should come fairly early in life.

In England, there is an ethical rule—how thoroughly enforced, we do not know—that a young physician must not enter into direct competition with established ones but must obtain a position as assistant, locum tenens or successor to some older doctor. To a certain extent, this principle is felt rather than expressed in small villages in this country. In a recent editorial, we have called attention to the fact that increased educational standards have so far diminished the graduation of physicians that the total is now only about 4,000, and that the mortality of the existing profession is about 3,000. With the increasing numbers and age of the profession and the continuance of the effect of educational standards, it will soon become quite feasible to exact such a requirement for the United States, though it is very doubtful whether it ever will be.

Still, it is obvious that there are very practical advantages for the young physician, in taking the place of an older man removed by death, or still better, by becoming his assistant and later his successor. Taking into consideration available government positions of various kinds, and the development or rapid increase of new settlements, it may be considered already possible for the recent graduate to fall into a position where he is really needed, rather than to jostle his predecessors to obtain standing room.

In Buffalo, during the last year, twenty-five physicians have either died, retired, or removed from the city. Scarcely a week passes but that, in our exchanges or by direct notification, we learn of the death of some practitioner in a neighboring villages; often leaving a community without convenient medical service. Another fact that has surprised us, is that the average county of the Empire State, contains about twenty post offices without a physician. As the government is very liberal in establishing post offices, this does not, of course, indicate that there is, in every case, a community lacking a physician. Still, there are few localities in the state except in the mountains, where the open country averages less than thirty persons to the square mile, and few roads which one can travel more than a couple of miles without passing a village or, at least a "Four-corners" which swells the average population materially. We are inclined to believe that a recent graduate, locating by chance in almost any one of the postoffices without a physician would find his average clientele of six hundred population, within a "ride" of four miles, though, of course, this "ride" might be overlapped by that of some established rival.

We have known, personally, of so many young physicians who have chosen village and country practices carefully, and with good success, that we cannot but feel that much of the complaint of overcrowding of our profession is due to lack of appreciation of local competitive factors, such as would receive prudent attention in regard to the location of any other business.

Even in the city, it is worth while to consider carefully the potential demands of a given district, not, of course, overlooking the fact that certain medical centers serve a comparatively large district.

We cannot refrain from adding a word of possible comfort to the unsuccessful physician in the city. We have known quite a number of such, whose lack of success was due to no specific cause but simply to the fact that, where a wide choice is possible, they, for some reason, were passed by for others. Among these, we have known a few who committed suicide, a few others who died pretty directly as the result of poverty—not that any one of them died literally of starvation or lack of clothing and shelter but because, in running the risks that all of us who had to earn their own living, felt that they must, these few succumbed, to pneumonia, grippe, phthisis, and the like. And we have known a few who sought a genuine demand for their services in the country, who found the physical strain of country practice by no means so arduous as it is depicted in novels, who found the fees not much less in theory and much more collectible in practice, beside having actually a far greater purchasing power than in the city. Under the stimulation of work which sought them instead of dodging their pursuit, they have developed self confidence and have waxed fat and prosperous, have been honored in their own locality and have enjoyed far greater success than they could ever have achieved in the city.

---

**Dr. Wiley** has not only been retained in his position, with an even firmer hold on the confidence of the profession and the people at large, but has proved that he had not even technically exceeded his authority nor entered into an arrangement to secure expert assistance at a higher rate than provided by law, except in a preliminary way, subject to official approval. The attempt to oust him has proved a boomerang, has developed support from honest manufacturers under control of the Food and Drug Law, and may eventually lead to far more stringent execution of the law, and on a more economic basis.

---

The Anti-Vaccinationists have issued a publication to thwart the efforts of the Buffalo Department of Health. No one can object to this method, providing, of course, that sincere and accurate arguments are used. We believe, personally, in the efficiency of vaccination against small pox, and, in an experience of about 5,000 cases, have never known small pox to develop after vaccination. In the 5,000 cases of vaccination, we have seen only one sore arm of serious degree and this was due to accidental infection with pyogens, under unfavorable surroundings, and it resulted in no permanent damage.

We believe that many anti-vaccinationists hold a mistaken impression of the attitude of the medical profession generally, in regard to vaccination, even charging mercenary motives. The most superficial study of facts suffices to disprove this assumption. Anti-variolar vaccination is about the only medical procedure in which the profession approves of free service to families of abundant means. To a large degree—amounting to about 95 per cent. of our personal experience—professional services are not even paid for at ordinary rates for salaried contracts but are required to be thrown in as good measure by physicians already working at the cheapest rates for municipalities, on board of ships, in the U. S. services, and the like. The relatively few vaccinations performed in private practice, represent the maximum of expense for materials used and the minimum of charges per visit. The profession believes, on statistic grounds of the most impressive and massive kind, that small pox can be prevented, with a minimum proportion of failures, by vaccination. Holding this view, if it were actuated in the slightest degree by mercenary motives, it would be the most foolish policy to urge vaccination. One case of small pox means more revenue to the medical profession than fifty vaccinations at ordinary private rates and more than it receives from 500 vaccinations as actually carried on.

Nor do we believe that the profession can be charged with being opinionated in the matter. We cannot conceive how any one who has made a careful study of statistics can doubt, either that the incidence and mortality of small pox have been reduced by vaccination to a degree representing the maximum of human perfection, nor that untoward accidents are adventitious and almost negligibly few in number. But no body of men are more eager to learn the truth, none more anxious for the general welfare, none more ready to acknowledge an error.

Anti-vaccination agitation may cause some trouble for health boards, but it will ultimately lead to the prevalence of truth and we are glad that it is assuming a tangible form where argument can be met with argument and statement with fact. We trust that we shall receive, for early publication, an exhaustive statistic and clinical study of this vital subject. Last year over 30,000 cases of small pox occurred in this country, the largest number for years. It is to be regretted that the compilation by the United States Public Health and Marine Hospital Service, could not have secured data as to previous vaccination. From the exceedingly low death rate, about 1½ per cent., we strongly suspect that most of the cases were of varioloid, and that they could have been prevented by repetition of vaccination. It is significant that the incidence varied from ½ to 4 or 5 cases per hundred thousand population in states where compulsory vaccination is practised and varied from fifty to two hundred and fifty per hundred thousand in states where one would naturally expect a large number of remotely vaccinated adults and where we may also suspect that school vaccination is not enforced.

It is a matter of history that, before vaccination was practised, small pox was about as prevalent and as fatal as scarlet fever, that is to say, it attacked pretty nearly everyone not in an isolated locality and it killed directly, about one case in ten. It is a safe inference that quarantine and general hygienic and sanitary measures would be about equally efficient for the two diseases. At present, scarlet fever kills, annually, about eleven persons per hundred thousand population. Small pox kills about one-sixth of one person per hundred thousand, in ordinary years, less than one-half per hundred thousand in 1910, a year of high incidence. IF THIS IS NOT DUE TO VACCINATION, TO WHAT IS IT DUE?

